# Management of newly diagnosed lupus nephritis in China: An implementation study based on the 2023 EULAR recommendations

**DOI:** 10.1007/s10067-025-07520-x

**Published:** 2025-06-07

**Authors:** Wei Bai, Yinli Gui, Jia Liu, Yunzhuan Chen, Xinwang Duan, Hongbin Li, Wei Wei, Lijun Wu, Shengyun Liu, Yanhong Wang, Shangyi Jin, Chanyuan Wu, Jiuliang Zhao, Qian Wang, Xiaomei Leng, Xinping Tian, Mengtao Li, Xiaofeng Zeng

**Affiliations:** 1 Department of Rheumatology and Clinical Immunology, Peking Union Medical College Hospital, Chinese Academy of Medical Sciences, Peking Union Medical College, National Clinical Research Center for Dermatologic and Immunologic Diseases (NCRC-DID), Ministry of Science & Technology, State Key Laboratory of Complex Severe and Rare Diseases, Peking Union Medical College Hospital, Key Laboratory of Rheumatology and Clinical Immunology, Ministry of Education, No.1 Shuaifuyuan, Dongcheng District, Beijing, 100730 China; 2 Department of Rheumatology, People’s Hospital of Zhengzhou: People’s Hospital of Henan University of Chinese Medicine, Henan Province, China; 3 Department of Rheumatology, Chaoyang Second Hospital, Liaoning Province, China; 4 Department of Rheumatology, The Second Affiliated Hospital of Nanchang University, Nanchang, Jiangxi Province China; 5 Department of Rheumatology and Immunology, The Affiliated Hospital of Inner Mongolia Medical University, Hohhot, Inner Mongolia Autonomous Region China; 6 Department of Rheumatology and Immunology, General Hospital of Tianjin Medical University, Tianjin, China; 7 Department of Rheumatology and Immunology, People’s Hospital of Xinjiang Uygur Autonomous Region, Urumqi, Xinjiang Uygur Autonomous Region China; 8 Department of Rheumatology and Immunology, The First Affiliated Hospital of Zhengzhou University, Zhengzhou, Henan Province China; 9 Department of Epidemiology and Bio-Statistics (YW), Institute of Basic Medical Science, Chinese Academy of Medical Science & Peking Union Medical College, Beijing, China

**Keywords:** European league against rheumatism recommendation, Immunosuppressant, Lupus nephritis, Standard of care, Systemic lupus erythematosus

## Abstract

**Background:**

Current evidence demonstrates that rigorous adherence to the standardized diagnostic and therapeutic protocols outlined in the European League Against Rheumatism (EULAR) guidelines can markedly enhance renal survival rates in patients with lupus nephritis (LN) and lead to improved overall outcomes. Data on the real-world implementation of LN management guideline in China was limited. This study aimed to examine the implementation of the 2023 EULAR recommendations for systemic lupus erymatosus (SLE) and LN in China.

**Methods:**

Based on the Chinese Systemic Lupus Erythematosus Treatment and Research Group (CSTAR) cohort, patients with newly diagnosed active LN (24-h Urine Protein ≥ 0.5 g/L) from April 2009 to March 2022 were included. The initial immunosuppressant therapy, the adjustment of immunosuppressant, and the implementation of renal biopsy at baseline were assessed. The patients’ general conditions, clinical manifestations, the condition of rheumatologists and hospitals were included in the analysis.

**Results:**

Of the 1518 enrolled patients, 787 (52%) underwent cyclophosphamide or mycophenolate mofetil as the initial immunosuppressant, which was consistent with the EULAR recommendations. It was associated with longer disease duration, higher disease activity, more mucocutaneous involvement, and younger age of onset. Thirty out of 166 patients (18.1%) failed to achieve renal remission after 6 or 12 months of treatment, and 9 of whom had immunosuppressive therapy adjusted as recommended. Overall, 251 patients (16.5%) underwent renal biopsy at baseline, which were associated with significant renal involvement, unremarkable systemic involvement, and treated by senior rheumatologists.

**Conclusions:**

Management of LN in China showed poor alignment with the 2023 EULAR recommendations regarding initial and adjusted therapy, as well as renal biopsy. The improvement of LN management requires both the patients and the rheumatologists to work together.

**Table:** 

**Key Points** • *To the best of our knowledge, this is the first real-world implementation study of LN in China. This is also the first real-world implementation study for the newly updated EULAR 2023 recommendation for SLE.* • *We not only examined the consistency between LN management in China and EULAR recommendations, but also identified potential factors associated with the implementation of the recommendations.* • *We believe that our study makes a significant contribution to the management of LN.*

## Introduction

Lupus nephritis (LN) is a severe organ involvement in systemic lupus erythematosus (SLE) caused by a massive deposition of immune complexes in the renal tissue[1]. The prevalence of renal involvement in SLE patients is approximately 50–60% in China[2], 40–80% in Asia[3], and 20–60% in Europe[4]. The cumulative incidence of progression to end-stage renal disease (ESRD) within 10 years in patients with LN is 10.1% in Asian people[5], among whom the rate of kidney flares (33–40%) was also higher than in other ethnic groups, showing a heavy economic and medical burden on both society and patients[3, 6, 7]. The Hopkins Lupus Cohort also had shown that lupus nephritis was both more frequent and more severe in East Asians than in African-Americans[8]. Although modern therapeutic strategies have successfully transformed LN from a life-threatening condition into a manageable chronic disease, disease recurrence continues to be a critical predictor of unfavorable prognosis. It has been shown that the longer the duration of clinical remission (sCR), the greater the likelihood of maintaining remission and the lower the risk of SLE disease recurrence[9]. Therefore, achieving and maintaining sCR should be considered a feasible goal in the management of LN to improve patients’ prognosis. The average annual direct medical costs of SLE patients varied significantly among different regions (ranging from $3,735 to $14,410)[10]. According to the results of a recent multicenter study in China, the average annual direct medical cost of LN patients in China is as high as 29,727 yuan, which is about 85% of the per capita disposable income of Chinese residents in 2021 (35,128 yuan)[11]. It is important to improve the management of LN because of its association with increased morbidity, mortality, and healthcare costs[12, 13].

Since the European League Against Rheumatism-European Renal Association-European Dialysis and Transplant Association (EULAR/ERA-EDTA) issued the first recommendation for LN management in 2012[14], several recommendations have been published in recent years, which have played a vital role in the clinical decision-making[15–19]. Updated evaluation and treatment strategies based on clinical manifestation and renal biopsy histopathology have significantly improved the management of LN. In the 2023 newly update EULAR recommendations for SLE, the treatment of active LN also received the highest attention[20]. However, despite the good quality of these recommendations, inadequate implementation in clinical practice may undermine their impact on LN management.

Previous studies have reported several problems regarding LN management, including delays in diagnosis and treatment, inadequate use of immunosuppressive agents, poor patient compliance, and high relapse rates[21]. Although previous study findings suggested potential inconsistencies between clinical practice and guidelines, there were limited data on the real-world implementation of recommendations in the patients with LN in China. Based on the Chinese Systemic Lupus Erythematosus Treatment and Research (CSTAR) group registry cohort, which is the largest registry of SLE in China, we aimed to examine the consistency between LN management in China and newly updated EULAR 2023 recommendations for SLE and LN, and to identify potential factors associated with the implementation of the recommendations.

## Methods

### Study population

This study is a retrospective cohort study based on real-world data. Patients with LN enrolled in the CSTAR cohort between April 2009 and March 2022. LN was defined as meeting the entry for"renal disease"in the classification criteria for SLE established by the ACR in 1997[22], SLICC/ACR in 2012[23], or EULAR/ACR in 2019[24]. Patients were eligible for inclusion if they satisfied all of the following criteria: (1) newly diagnosed with LN (with a time interval of less than 3 months between LN diagnosis and registration), (2) baseline 24-h urine protein (24-hUP) level of ≥ 0.5 g, and (3) complete baseline treatment records. LN patients included in the immunosuppressant adjustment implementation study must complete at least one follow-up record within 6 or 12 months. (Patients with a follow-up time deviation of less than 1 month from the specified time are also eligible for inclusion). Patients with missing core data are routinely identified and excluded from the database. Before enrollment, written informed consent was obtained from all patients or their legal guardians.

### Implementation analysis

We assessed the implementation of recommended care in the study population based on the following key strategies: (1) initial immunosuppression therapy was initiated in patients with LN immediately upon baseline registration, (2) adjustment of immunosuppression during follow-up (at 6 months or 12 months) in patients who failed to achieve treatment goals, and (3) implementation of renal biopsy in newly diagnosed patients with LN at baseline.

#### Initial therapy

First, patients were divided into two groups according to the immunosuppressants for intial treatment as follows: (1) cyclophosphamide (CYC) or mycophenolate mofetil (MMF) alone, combined with glucorticoids as the initial therapy, which was in concordance with the 2023 EULAR recommendation for initial induction treatment in patients with active LN; (2) other immunosuppressant therapies, such as cyclosporine A (CsA), tacrolimus (Tac), azathioprine (AZA), leflunomide (LEF) and sirolimus (SRL), combination of two or more immunosuppressive agents, and therapies without any immunosuppressive agent.

#### Adjustment of immunosuppressant

Patients who initiated CYC or MMF monotherapy were further followed up for 6 to 12 months to find out whether the immunosuppressive agents were adjusted in non-responders. According to the EULAR recommendations, if levels of proteinuria in patients with active LN did not decrease by 50% within 6 months, or urine protein-creatine ratio (UPCR) remained to be above 500–700 mg/g after 12 months, the initial treatment strategy was considered to be failed, and prompt adjustment of immunosuppression should be performed. Therefore, in accordance with the recommendations of the guidelines, the adjusted immunosuppressive regimens include the following scenarios: from CTX to MMF, from MMF to CTX, or from CTX/MMF to RTX. All of these are regarded as treatment adjustments that comply with the requirements of the guidelines.

#### Implementation of renal biopsy at baseline

Patients with LN were divided into two groups according to whether a renal biopsy was performed at baseline. Based on the 2019 EULAR recommendations for LN[15], renal biopsy should be considered when there is evidence of kidney involvement. The group with patients who underwent renal biopsy at baseline was thought to be consistent with the EULAR recommendations.

### Statistical analysis

Descriptive statistics were used to present the demographic and clinical characteristics of the patients and information about the providers and hospitals. Normally distributed continuous variables are presented as means and standard deviations, non-normally distributed variables are presented as medians and interquartile ranges, and categorical variables are presented as rates. Student’s t-test and Mann–Whitney U test were used to analyze continuous variables, and the chi-squared test was used to analyze categorical variables. All statistical analyses were performed using SPSS software (version 23.0; IBM SPSS Inc., Armonk, NY, USA). Statistical significance was defined as P < 0.05.

## Results

### Factors associated with concordant with the 2023 EULAR recommendations regarding initial therapy

Overall, 1518 patients with active LN were included in this study. At baseline, 1019 (67.1%) patients initiated immunosuppressive agents, of which 931 (61.3%) administered immunosuppressive monotherapy. Furthermore, CYC was used in 442 patients (29.1%), followed by MMF in 345 patients (22.7%), Tac in 57 patients (3.8%), CsA in 43 patients (2.8%), LEF in 30 patients (2.0%), SRL in 5 patients and AZA in 4 patients. Combinations of two immunosuppressive agents were given to 88 patients (5.8%), including CYC + MMF in 27 cases (1.8%), MMF + Tac in 24 cases (1.6%), CYC + Tac in 9 cases (0.6%), CYC + LEF in 8 cases, CYC + CsA in 6 cases, and MMF + CsA in 3 cases. There were two cases each of CYC + rituximab (RTX), MMF + LEF, and MMF + RTX, and one case each of Tac + LEF, RTX + LEF, CYC + SRL, MMF + SRL, and CsA + LEF.

Of the 1518 patients, 787 (51.8%) were in the guideline-compliant group (those who met the guidelines and received CYC or MMF alone as immunosuppressant for intial treatment), and 731 (48.2%) were in the non-guideline-compliant group (those who did not receive CYC or MMF alone). Patients in the guideline-compliant group had a younger age of onset (34.7 ± 13.8 years vs. 36.6 ± 14.6 years, *P* = 0.023), longer disease duration [3.00 (1.00, 12.00) months vs. 2.00 (1.00, 9.00) months, *P* = 0.019], higher SLEDAI scores (14.9 ± 7.2 vs. 13.9 ± 7.4, *P* = 0.005) and PGA scores [2.0 (1.5, 2.4) vs. 1.9 (1.4, 2.2), *P* = 0.007], higher incidence of hypocomplementemia (85.0% vs. 76.5%, *P* < 0.001), higher incidence of mucocutaneous involvement (45.1% vs. 38.3%, *P* = 0.007), and a higher rate of anti-ds-DNA positivity (71.7% vs. 65.7%, *P* = 0.012) than patients in the non-guideline compliant group.

There were no significant differences between the two groups regarding hospital and physician related factors, sex, education, medical insurance, proportion of renal biopsy, SDI score, other clinical manifestations, 24-hUP, serum creatinine, and albumin levels (All *p* values were ≥ 0.05) (Table 1).

**Table 1:** Comparison of clinical and laboratory indicators in 1518 patients with active LN who received an initial immunosuppressive prescription

Variables	Monotherapy CTX/MMF (guideline-compliant group) (*N* = 787)	Non-monotherapy CTX/MMF or no IS added (non-guideline-compliant group) (*N* = 731)	*P* value
Characteristics			
Female sex [n (%)]	677 (86.0)	640 (87.6)	0.38
Age (years), mean ± SD	34.7 ± 13.8	36.6 ± 14.6	0.023
Education (bachelor's degree and above) (%)	18.0 (88/488)	14.8 (59/398)	0.202
Medical insurance (no insurance) (%)	18.5(91/492)	22.3 (89/400)	0.165
Disease duration (months), median (interquartile range)	3.00 (1.00–12.00)	2.00 (1.00–9.00)	0.019
Renal biopsy [n (%)]	142 (18.0)	109 (14.9)	0.101
SLEDAI score, mean ± SD	14.9 ± 7.2	13.9 ± 7.4	0.005
PGA Score, median (interquartile range)	2.0 (1.5–2.4)	1.9(1.4–2.2)	0.007
SDI score, median (interquartile range)	0.00 (0.00–1.00)	0.00 (0.00–1.00)	0.105
Laboratory data			
Hypocomplementemia [n (%)]	669 (85.0)	559 (76.5)	< 0.001
Positive anti-dsDNA [n (%)]	564 (71.7)	480 (65.7)	0.012
24-hUP (g/L), median (interquartile range)	2.00 (1.00–4.08)	1.81 (0.95–3.65)	0.103
Serum creatinine (μmol/L), median (interquartile range)	65.8 (51.0–91.0)	67.0 (52.2–92.6)	0.504
Serum albumin (g/L), mean ± SD	28.5 ± 6.9	28.6 ± 7.2	0.875
Clinical manifestations			
Vasculitis [n (%)]	67 (8.5)	60 (8.2)	0.83
Arthritis [n (%)]	216 (27.4)	189 (25.9)	0.484
Myositis [n (%)]	28 (3.6)	25 (3.4)	0.884
Serositis [n (%)]	183 (23.3)	166 (22.7)	0.801
Mucocutaneous involvement [n (%)]	355 (45.1)	280 (38.3)	0.007
Fever [n (%)]	156 (19.8)	127 (17.4)	0.221
Thrombocytopenia [n (%)]	211 (27.7)	222 (30.4)	0.256
Leukopenia [n (%)]	244 (31.0)	234 (32.0)	0.673
Neuropsychiatric lupus [n (%)]	63 (8.0)	46 (6.3)	0.197
Comorbidities			
Diabetes [n (%)]	9 (1.1)	16 (2.2)	0.11
Hypertension [n (%)]	88 (11.2)	72 (9.8)	0.398
Hospital and physician			
Tertiary hospital [n (%)]	784 (99.6)	729 (99.7)	0.715
Female physicians (%)	48.6(298/613)	54.1(320/591)	0.055
Physician title (senior level) [n (%)]	415 (52.7)	397(54.3)	0.538
Years of physician practice (years), mean ± SD	14.4 ± 8.3	15.0 ± 8.8	0.444

Values are presented as mean ± SD, n (%), and median (Q1–Q3). *IS* Immunosuppressant, *SLEDAI* SLE Disease Activity Index, *PGA* Physician’s Global Assessment, *SDI* Systemic Lupus International Collaborating Clinics/American College of Rheumatology Damage Index, *dsDNA* double-stranded DNA, *24-hUP* 24-h Urine Protein

### Factors associated with concordant with the 2023 EULAR recommendations regarding adjustment of immunosuppressant

Of the 787 patients in the guideline-compliant group mentioned above, 166 patients had complete 6- or 12-month follow-up data. After 6 or 12 months of treatment, 30 patients (30/166, 18.1%) failed to achieve the target, and 9 (9/30, 30%) of them adjusted the immunosuppressant following the guideline, and the other 21 patients did not.

The two groups of patients who were failure to initial therapy had no statistically significant differences regarding general condition, clinical manifestations, laboratory data, treatment hospital, or physician-related factors (All *p* values were ≥ 0.05) (Table 2).

**Table 2:** Comparison of clinical and laboratory indices in LN patients failed to initial treatment with an adjusted immunosuppression regimen

Variables	Immunosuppressant adjustment group(*N* = 9)	Immunosuppressant unadjusted group(*N* = 21)	*P* value
Characteristics			
Female sex [n (%)]	8 (88.9)	20 (95.2)	0.523
Age (years), mean ± SD	34.1 ± 11.6	33.8 ± 13.6	0.947
Education (bachelor's degree and above) (%)	12.5 (1/8)	21.2 (3/14)	0.601
Medical insurance (no insurance) (%)	37.5 (3/8)	14.3 (2/14)	0.211
Disease duration (months), median (inter-quartile range)	4.00 (0.00–7.00)	5.00(1.00–25.00)	0.227
Renal biopsy [n (%)]	1 (12.5)	5 (23.8)	0.502
SLEDAI score, mean ± SD	18.2 ± 10.2	15.2 ± 6.3	0.333
PGA Score, median (interquartile range)	2.00 (0.40–2.75)	2.00 (1.28–2.00)	0.867
SLICC/ACR damage index score, median (inter-quartile range)	0.00 (0.00–1.00)	0.00(0.00–0.00)	0.509
Laboratory data			
Hypocomplementemia[n (%)]	8 (88.9)	20 (95.2)	0.523
Positive anti-dsDNA [n (%)]	8 (88.9)	13 (61.9)	0.139
24-hUP (g/L), median (inter-quartile range)	4.52 (4.08–10.40)	2.57(1.31–7.83)	0.213
Serum creatinine (μmol/L), median (interquartile range)	80.7 (58.5–139.0)	60.0(43.0–90.9)	0.151
Serum albumin (g/L), mean ± SD	22.5 ± 5.0	27.0 ± 7.2	0.151
Clinical manifestations			
Vasculitis [n (%)]	2 (22.2)	1 (4.8)	0.144
Arthritis [n (%)]	4 (44.4)	4(19.0)	0.149
Myositis [n (%)]	0	0	NA
Serositis [n (%)]	3 (33.3)	4 (19.0)	0.397
Mucocutaneous involvement [n (%)]	1 (11.1)	10 (47.6)	0.057
Fever [n (%)]	2 (22.2)	4 (19.0)	0.842
Thrombocytopenia [n (%)]	1 (11.1)	5 (23.8)	0.426
Leukopenia [n (%)]	1 (11.1)	3 (14.3)	0.815
Neuropsychiatric lupus [n (%)]	1 (11.1)	4 (19.0)	0.593
Comorbidities			
Diabetes [n (%)]	1 (11.1)	0 (0)	0.12
Hypertension [n (%)]	0	0	NA
Hospital and physician			
Tertiary hospital [n (%)]	9 (100)	21 (100)	NA
Female physicians (%)	57.1%(4/7)	50%(9/18)	0.748
Physician title (senior level) [n(%)]	6 (66.7)	13 (61.9)	0.804
Years of Physician Practice (years), mean ± SD	15.0 ± 9.5	17.8 ± 6.5	0.415

Values are presented as mean ± SD, n (%), and median (Q1–Q3). *IS* Immunosuppressant, *SLEDAI* SLE Disease Activity Index, *PGA* Physician’s Global Assessment, *SDI* Systemic Lupus International Collaborating Clinics/American College of Rheumatology Damage Index, *dsDNA* double-stranded DNA, *24-hUP* 24-h Urine Protein

### Factors associated with concordant with the EULAR recommendations regarding renal biopsy

Of the 1518 enrolled patients, 251 (251/1518, 16.5%) underwent renal biopsies at baseline, which was considered to be consistent with the recommendations. Compared with patients who did not have renal biopsy, these patients had higher 24-hUP quantification [2.99 g/L (1.42, 5.68) vs. 1.75 g/L (0.90, 3.60), *P* < 0.001], higher serum creatinine levels [70.8 μmol/L (55.0, 100.0) vs. 65.6 μmol/L (51.0, 90.6), *P* = 0.007], higher incidence of hypertension (14.7% vs. 9.7%, *P* = 0.018), and lower serum albumin levels (27.5 ± 7.7 g/L vs. 28.7 ± 6.9 g/L, *P* = 0.02), SLEDAI scores (13.6 ± 7.0 vs. 14.6 ± 7.3, *P* = 0.017), and PGA scores [1.80 (1.20, 2.10) vs. 2.00 (1.50, 2.30), *P* = 0.007]. Compared with patients who did not undergo renal biopsy, these patients had lower incidence of fever (12.4% vs. 19.9%, *P* = 0.005), serositis(15.9% vs. 24.4%, *P* = 0.004), mucocutaneous involvement (35.9% vs. 43.0%, *P* = 0.036), thrombocytopenia (20.3% vs. 30.1%, *P* = 0.002), and leukopenia (24.3% vs. 32.9%, *P* = 0.007).

Physicians in the guideline-compliant renal biopsy group had longer years of practice (16.7 ± 9.8 years vs. 14.3 ± 8.3 years, *P* = 0.003). There was no significant difference between the two groups regarding compliance with the guidelines for initial immunosuppressive therapy (56.6% vs. 50.9%,* P* = 0.101). In terms of compliance with the the EULAR recommendations regarding renal biopsy, there were no significant differences in gender (*P* = 0.887) or professional titles (*P* = 0.705) between the two groups of doctors (Table 3).

**Table 3:** Comparison of clinical laboratory indices and immunosuppressive prescriptions between two groups of 1518 active LN with/without renal biopsy

Variables	renal biopsy group (guideline-compliant group) (*N* = 251)	Non-renal biopsy group(non-guideline compliant group) (*N* = 1267)	*P* value
Characteristics			
Female sex [n (%)]	214 (85.3)	1103 (87.1)	0.443
Age (years), mean ± SD	34.6 ± 13.4	35.8 ± 14.3	0.27
Education (bachelor's degree and above) (%)	19.3 (31/161)	16.0 (116/725)	0.315
Medical insurance (no insurance) (%)	19.4 (31/160)	20.4 (149/732)	0.78
Disease duration (months), median (inter-quartile range)	2.00 (1.00–9.00)	3.00 (1.00–12.00)	0.546
SLEDAI score, mean ± SD	13.6 ± 7.0	14.6 ± 7.3	0.017
PGA Score, median (range)	1.80 (1.20–2.10)	2.00 (1.50–2.30)	0.017
SLICC/ACR damage index score, median (inter-quartile range)	0.00 (0.00–1.00)	0.00 (0.00–1.00)	0.286
Compliance with guidelines initial IS prescription [n (%)]	142 (56.6)	645 (50.9)	0.101
Laboratory data			
Hypocomplementemia [n (%)]	194 (77.3)	1034 (81.6)	0.112
Positive anti-dsDNA [n (%)]	164 (65.3)	880 (69.5)	0.199
24-hUP (g/L), median (interquartile range)	2.99 (1.42–5.68)	1.75 (0.90–3.60)	< 0.001
Serum creatinine (μmol/L), median (inter-quartile range)	70.8 (55.0–100.0)	65.6 (51.0–90.6)	0.007
Serum albumin (g/L), mean ± SD	27.5 ± 7.7	28.7 ± 6.9	0.02
Clinical manifestations			
Vasculitis [n (%)]	17 (6.8)	110 (8.7)	0.318
Arthritis [n (%)]	62 (24.7)	343 (27.1)	0.438
Myositis [n (%)]	6 (2.4)	47 (3.7)	0.298
Serositis [n (%)]	40 (15.9)	309 (24.4)	0.004
Mucocutaneous involvement [n (%)]	90 (35.9)	545 (43.0)	0.036
Fever [n (%)]	31 (12.4)	252 (19.9)	0.005
Thrombocytopenia [n (%)]	51 (20.3)	382 (30.1)	0.002
Leukopenia [n (%)]	61(24.3)	417 (32.9)	0.007
Neuropsychiatric lupus [n (%)]	11 (4.4)	98 (7.7)	0.06
Comorbidities			
Diabetes [n (%)]	5 (2.0)	20 (1.6)	0.638
Hypertension [n (%)]	37 (14.7)	123 (9.7)	0.018
Hospital and physician			
Tertiary hospital [n (%)]	249 (99.2)	1264 (99.8)	0.157
Female physicians (%)	51.8 (101/195)	51.2 (517/1009)	0.887
Physician title (senior level) [n (%)]	137 (54.6)	675 (53.3)	0.705
Years of physician Practice (years), mean ± SD	16.7 ± 9.8	14.3 ± 8.3	0.003

Values are presented as mean ± SD, n (%), and median (Q1–Q3). *IS* Immunosuppressant, *SLEDAI* SLE Disease Activity Index, *PGA* Physician’s Global Assessment, *SDI* Systemic Lupus International Collaborating Clinics/American College of Rheumatology Damage Index, *dsDNA* double-stranded DNA, *24-hUP* 24-h Urine Protein

## Discussion

Based on the CSTAR study cohort, we firstly evaluated the implementation of the 2023 EULAR recommendations for SLE and LN in China, including choice of initial immunosuppressive therapy, adjustment after failure of the first treatment stragety, and the implementation of renal biopsy, referring to the 2019 EULAR recommendations for LN. To our knowledge, this is the first real-world implementation study for the recommendations for LN and the newly updated EULAR 2023 recommendations for SLE.

LN is incurable but manageable, and patients need long-term medication to suppress disease activity, prevent flares, and slow progression. Disease flares significantly increase the risk of organ damage in patients with systemic lupus erythematosus (SLE), and cumulative damage may increase the likelihood of new injuries and mortality[25]. The rising healthcare costs associated with LN treatment urgently require in-depth research and standardization of SLE treatment and management strategies to effectively prevent relapses and limit progression, and to reduce the burden of disease. Similar to out study, based on Chinese Rheumatism Data Center (CRDC) data, a study on the current status of implementation of rheumatoid arthritis (RA) management in China published in 2022 suggest a significant gap between recommended treatment regimens for RA and clinical practice [26]. The formation of this gap is influenced by a variety of factors, including incomplete understanding of treatment recommendations, concerns about drug safety and financial burden, and patient adherence issues, all of which have a significant impact on clinicians'decision-making. Thus, we further conducted a clinical practice study for patients with newly diagnosed LN, evaluated the practical application of the 2023 EULAR guidelines in China, and analyzed the major factors that hinder guideline adherence, aiming to provide new solutions and references to improve the management of LN in China.

Since 2012, several recommendations for the management and treat-to-target strategy of LN had been established by many official international association organizations[27], such as ACR[28], EULAR[17], the Kidney Disease: Improving Global Outcomes (KDIGO)[16], and Chinese Rheumatology Association[29]. Although these guidelines are based on different research backgrounds and population scopes, the use of MMF or CYC during the induction therapy phase is always considered to be the standard of care (SoC) for patients with LN. In the 2023 update of EULAR management recommendation of SLE[20], combination therapy with belimumab (either with CYC or MMF) or calcineurin inhibitors (especially voclosporin or TAC, combined with MMF) were considered as an intial treatment option for active LN. However, in our study, we still considered the use of MMF or CYC alone as the therapy compliant with the EULAR recommendations. It was because that even in the 2023 updated EULAR recommendation, the combination therapy was still controversial, compared to the SoC therapy (MMF or CYC alone) in LN (the evidence level: 1b/A *vs.* 1a/A). Moreover, as shown in the results, the proportion of patients treated by multi-target therapy (MMF + Tac) in our study was also relatively low (less than 2%). That’s why we still focused on the implementation of classical SoC therapy in our study.

Previous studies had shown the implementation of SoC therapy in the management of active LN. Laura Bartels-Peculis et al.[30] had conducted a retrospective observational study using a health insurance plan database. The analysis of 1039 patients with LN showed that 41% of patients received immunosuppressive therapy, which was lower than the result of our study cohort. In the initial SLICC cohort[5], the initial standard immunosuppressant treatment rate among LN patients was 70.5%, which was higher than 51.8% in our cohort. In another multicenter, real-world observational study focusing on immunosuppressive regimens and treatment adjustments, the initial prescription rate of immunosuppressants among LN patients was reached 83.6%[31]. However, in our study there were also approximately 1/3 of the patients who were not treated with immunosuppressants (mostly with glucocorticoids alone, not shown in the results) as the initial treatment for LN, and approximately half of the patients who were not treated with immunosuppressants in concordance with the EULAR recommendation (MMF or CYC alone). These results indicated low awareness of standard immunosuppressive therapy during the initial treatment phase of LN in China. Concordance of initial treatment with EULAR recommendations was associated with longer disease duration and more typical and severe disease activity, such as more mucocutaneous involvement, higher rates of hypocomplementemia and anti ds-DNA antibody, and higher SLEDAI scores and PGA scores. It seemed like more severe patients had better adherence to SoC. Interestingly, we observed that younger patients were more likely treated by immunosuppressants in concordance with guideline recommendations. Treatment options were not significantly correlated with the practitioner's title, years of practice, or sex, suggesting that poor awareness of standardized management in accordance with the guideline recommendations was a common problem.

Studies have confirmed that early response to treatment and rapid achievement of complete renal remission in patients with LN is strongly associated with better long-term renal prognosis[32–34]. Additional studies have shown that early diagnosis and standard of care are important for maintaining long-term stability of renal function and improving the quality of life of patients with LN[35–37]. However, in real-world clinical practice, the low rate of complete remission and the risk of drug adverse effects were still major problems in LN treatment[38]. In this study, among the patients who had poor responses to initial treatment, less than one third of them adjusted the immunosuppressant following the recommendation. That might because of both the patients and the physicians had a poor adherence with the guideline if the initial treatment was failed to achieve treatment target at beginning. On the other hand, the fact that only 18.1% (30/166) patients had poor response to initial standard of care therapy seemed to demonstrated the effectiveness of treatment strategy recommended by guidelines. In our cohort, 18.1% of the patients showed poor response to the initial immunosuppressive (IS) therapy and thus required adjustment of the treatment plan. This result is consistent with the real-world data reported by Pappa M et al. from a multi-center rheumatology department in Greece, which indicated that 84.2% of newly diagnosed lupus nephritis (LN) patients responded well to the standard of care (SoC) regimen[31]. It is also in line with the findings from a large LN cohort study conducted in Italy[39], which reported an overall remission rate of 84%. However, a recent report released by the Lupus Network of the Accelerated Access to Medicines Partnership (AMP) in the United States shows that the overall remission rate at one year is only 44%, which is highly consistent with the data from randomized clinical trials[40].

Renal histopathology had been considered as the gold standard for diagnosing LN as shown in the classification criteria of SLE. Approximately 90% of patients with SLE exhibit pathological manifestations of LN when they undergo renal biopsy[41]. Inconsistencies between the clinical manifestations of LN and renal pathology indicate that the pathological type of LN plays a pivotal role in evaluating disease activity, guiding treatment selection and outcome prediction. Recently, major guidelines have emphasized the importance of renal biopsy pathology[15, 16]. In our study, the proportion of patients with active LN who underwent renal biopsy according to the guidelines was only 16.5%, compared with 48.8% of patients who underwent renal biopsy at the time of the first suspicion of nephritis in the SLICC's initial LN cohort[5]. We analyzed the reasons for the low renal biopsy may include: 1. The CSTAR cohort mainly conducted by rheumatologists, whom may have less experience in renal puncture biopsy than nephrologists. Patients from department of Rheumatology may have concurrent multi-systemic involvement with contraindications to renal puncture, which may also affect the performance of renal puncture. It was demonstrated in our study that patients in the non-renal biopsy group had milder renal involvement but relatively more severe systemic involvement. 2. Inadequate healthcare capacity. In this study, approximately one fifth of the patients lacked insurance coverage. Under the fee-for-service healthcare system, these patients may have faced underinsurance issues, which potentially deterred them from undergoing renal biopsy. This could have influenced the overall implementation of renal biopsies. 3. Patient's personal factors refused to undergo the test. In this regard, in the future, we expect to rely on the Chinese Rheumatism Data Center(CRDC)[42], fully leverage the leading role of the National Clinical Research Center for Medical Sciences, build a nationwide collaborative network for specialized disease research, strengthen collaboration among relevant specialties, promote discipline construction and development, enhance doctors'awareness of standardized diagnosis and treatment, and thereby comprehensively improve the overall research level and clinical practice ability of SLE in China.

The low concordance with the implementation of the EULAR guideline recommendations may mean that Chinese patients with SLE may face the risk of higher disease recurrence rates, accumulation of more organ involvement and increased side effects associated with therapeutic drugs in clinical practice, and the concept of standardized treatment for SLE is challenged. These differences may be related to patients'weak awareness of standardized treatment and regular follow-up, socioeconomic factors, primary care physicians'insufficient knowledge of the disease in SLE, the accessibility of medications, and insufficient knowledge of standardized and up-to-date treatment among some medical professionals. It has been reported that only 0.76% of SLE patients in China are able to achieve complete remission of the disease without medication, and the disease and economic burden caused by SLE is increasing[43]. The results of a study conducted by the Department of Rheumatology and Immunology at Peking Union Medical College Hospital and the School of Public Health at Peking University using the 2013–2017 National Urban Workers'and Residents'Basic Medical Insurance Database showed that the prevalence of SLE in China's urban population increased 1.18-fold in 5 years, and the overall and per capita annual medical costs for SLE patients were 144.5 billion dollars and 1599.34 dollars, respectively[44]. Overall, the increasing number of SLE patients and the huge disease expenditures have created a growing individual and social health burden in China.

It was worth noting that in our study we included SLE patients in CSTAR cohort established in 2009, which might have influence on the results because of the earlier the patients were involved, the more likely treatment irregularities were present. In fact, during these years, the management of SLE in China has been rapidly improved. In 2019, the National Health Commission of the People's Republic of China has released the Guidelines for the Construction and Management of Departments of Rheumatology in General Hospitals and the Guidelines for the Basic Standards of Department of Rheumatology in General Hospitals, which lays the groundwork for further initiation of the developing Centers of Excellence (COEs) for better management of SLE patients in China[45]. We’ve established the grading system for COEs to promote the standard-of-care in lupus, including disease evaluation, early intervention, adherent to Treat-to-target (T2 T) strategy and regularly monitor comorbidities and complications in SLE. COEs should also provide physician training to local rheumatologists and establish a multi-disciplinary team (MDT) to promote the implementation of standard-of-care. These approaches can lead to better compliance with guidelines for standardized treatment in future.

This study has some limitations. First, the CSTAR study center was dominated by rheumatologists in tertiary care hospitals and lacked data from patients in primary care hospitals, which may have led to physician and patient selection bias. Patients with LN treated by nephrologists may have received more renal biopsies, and there may be a potential discrepancy between this and actual implementation by rheumatologists according to renal biopsy guidelines. Second, only a relatively small number of patients in our study cohort had complete 6- or 12-month follow-up data, which may had an impact on the results of the study of the implementation of immunosuppression adjustments. In the future, further studies involving a large number of patients with regular follow-up data are needed to provide more convincing data on nonresponding or refractory cases in real-world practice. Third, because the CSTAR study cohort was established in 2009, the proportion of patients receiving novel therapies was relatively low in these years, and data on novel therapies in LN, particularly belimumab and novel calcineurin inhibitors (CNIs), should be updated in future studies. Despite these limitations, this study provides data on the implementation of standard therapies in Chinese patients with LN following guideline recommendations in the real world and summarizes the characteristics of patients with LN who follow guideline recommendations for implementation, as well as identifying useful information such as potential factors associated with implementation of recommendations.

## Conclusions

This study provided the implementation of Chinece LN management in accordance with 2023 EULAR recommendations for SLE and LN. It showed that Chinese patients with LN had low awareness of standard immunosuppressive therapy during induction therapy phase, especially elder patients, and patients with milder disease activity and shorter disease duration. In patients who were failure to achieve the treatment goals after initial treatment, the proportion of adjust immunosuppressant following the guideline recommendation was also lower than expect. Patients who underwent renal biopsy in accordance with guideline usually had significant renal involvement and milder systemic involvement. Senior physicians were more likely to provide renal biopsies according to the guidelines. In future, the implementation of standard-of-care in SLE and LN may be improved by the development of Rheumatology in China, for example, the construction of more COEs to strengthen multidisciplinary cooperation and improve the adherence of patients.

## Data Availability

The data that support the findings of this study are available on request from the corresponding author (ML or XZ), upon reasonable request.

## Declarations

### Patient and public involvement

Patients or the public were not involved in the design, or conduct, or reporting, or dissemination plans of our research.
